# Probiotics (*Bifidobacterium longum*) Increase Bone Mass Density and Upregulate *Sparc* and *Bmp-2* Genes in Rats with Bone Loss Resulting from Ovariectomy

**DOI:** 10.1155/2015/897639

**Published:** 2015-08-20

**Authors:** Kolsoom Parvaneh, Mahdi Ebrahimi, Mohd Redzwan Sabran, Golgis Karimi, Angela Ng Min Hwei, Saif Abdul-Majeed, Zuraini Ahmad, Zuriati Ibrahim, Rosita Jamaluddin

**Affiliations:** ^1^Department of Nutrition and Dietetics, Faculty of Medicine and Health Sciences, Universiti Putra Malaysia, 43400 Serdang, Selangor, Malaysia; ^2^Department of Veterinary Preclinical Sciences, Faculty of Veterinary Medicine, Universiti Putra Malaysia, 43400 Serdang, Selangor, Malaysia; ^3^Tissue Engineering Center, Faculty of Medicine, Universiti Kebangsaan Malaysia, 50300 Kuala Lumpur, Malaysia; ^4^Department of Life Sciences, School of Pharmacy, International Medical University, No. 126, Jalan Jalil Perkasa 19, Bukit Jalil, 57000 Kuala Lumpur, Malaysia; ^5^Department of Biomedical Science, Faculty of Medicine and Health Sciences, Universiti Putra Malaysia, 43400 Serdang, Selangor, Malaysia

## Abstract

Probiotics are live microorganisms that exert beneficial effects on the host, when administered in adequate amounts. Mostly, probiotics affect the gastrointestinal (GI) tract of the host and alter the composition of gut microbiota. Nowadays, the incidence of hip fractures due to osteoporosis is increasing worldwide. Ovariectomized (OVX) rats have fragile bone due to estrogen deficiency and mimic the menopausal conditions in women. Therefore, this study aimed to examine the effects of *Bifidobacterium longum* (*B. longum*) on bone mass density (BMD), bone mineral content (BMC), bone remodeling, bone structure, and gene expression in OVX rats. The rats were randomly assigned into 3 groups (sham, OVX, and the OVX group supplemented with 1 mL of *B. longum* 10^8^–10^9^ colony forming units (CFU)/mL). *B. longum* was given once daily for 16 weeks, starting from 2 weeks after the surgery. The *B. longum* supplementation increased (*p* < 0.05) serum osteocalcin (OC) and osteoblasts, bone formation parameters, and decreased serum C-terminal telopeptide (CTX) and osteoclasts, bone resorption parameters. It also altered the microstructure of the femur. Consequently, it increased BMD by increasing (*p* < 0.05) the expression of *Sparc* and *Bmp-2* genes. *B. longum* alleviated bone loss in OVX rats and enhanced BMD by decreasing bone resorption and increasing bone formation.

## 1. Introduction

Osteoporosis is a bone metabolic disorder, which is explained by low bone mass and deterioration of the bone tissue, leading to increase in bone weakness. Due to osteoporosis, bone density and microarchitecture of the bone tissue are decreased, leading to the risk of fracture [1]. The incidence of hip fractures due to osteoporosis is increasing worldwide and the elderly population is mostly affected by the risk of fracture [2]. The total disability adjusted life years were reported at 5.8 million worldwide. Fifty-one percent of this number was a result of fractures, which occurred mostly in population from Europe and America. Thus, osteoporotic fractures were reported as an important cause of mortality and morbidity specifically in developed countries [3]. Indeed, the most common type of osteoporosis is associated with postmenopausal conditions among women aged 50 years and over [4].

One-third of women lifespan is spent during the menopausal period [5]. Thus, it is necessary to reduce the adverse effect of menopause on women health. Hormone replacement therapy (HRT) was shown to reduce the risk of fracture among patients with osteoporosis. However, due to its daily injection, most women are not keen on this treatment. Moreover, HRT increases the possibility of tumorigenesis [6]. Therefore, the identification of alternative ways to increase bone formation and maintain the bone strength is considered vital, especially for menopausal women.

A possible osteoporosis treatment is the consumption of probiotics [7, 8]. Probiotics are live microorganisms [9], sometimes derived from fermented food [10], which confer a beneficial physiological effect on the host when administered in adequate amounts [9, 11]. Recently, few studies reported that probiotics can decrease the incidence of osteoporosis [8, 12, 13]. Some strains of probiotics are investigated for their effects on osteoporosis such as* Lactobacillus casei*,* Lactobacillus plantarum*,* Lactobacillus paracasei,* and* Bifidobacterium longum* (*B. longum*).

Recently, the important function of gut microbiota (GM) in the treatment of different diseases, including bone loss, was studied. The GM comprises millions of bacteria and can be modulated with some environmental factors such as diet. The absence of GM in germ-free mice increased bone loss. However, colonization of the germ-free mice with a normal GM increased immunity and bone mass [14]. Probiotics mostly act by changing the composition of the GM [15] and increase the solubility and absorption of minerals, which lead to the modulation of the immune system [16, 17]. The bone remodeling process is one system modulated by probiotics [12].

The regulation of bone metabolism is complex and some factors such as genetics, the environment, and lifestyle contribute to the bone metabolism [18]. In addition, genetic factors play a vital role in bone turnover, which have accounted for 70 to 80% of variation in bone density [19]. The cells that influence bone density are osteoblasts, osteoclasts, osteocytes, and lining cells with their own structure and role. These cells are mainly affected by probiotics during the bone remodeling process [1]. Osteoclasts are large, multinucleated cells associated with osteoclastogenesis and are responsible for bone resorption. Osteoblasts are mononuclear bone formation cells and are responsible for the bone formation and mineralization. Osteoid is located on the bone surface and plays a role in the mechanical stimulation and initiation of the remodeling response [20].

Ovariectomy in rats causes bone loss [21]. OVX is defined as the surgical removal of one or both ovaries. This term is usually used in basic science studies to explain the removal of ovaries in laboratory animals, including rodents. OVX leads to the loss of ability to secrete estrogen and progesterone. Besides, the surgery also decreases testosterone production and leads to a condition known as surgical menopause. Thus, with the OVX surgical process, sexual hormones are decreased, followed by a rise in bone loss and the risk of bone fractures, which are similar to menopausal osteoporotic conditions in women.

To the best of our knowledge, no study has been conducted and published on the effect of* B. longum* in OVX rats. Therefore, the aim of this study was to examine the effectiveness of the probiotic,* B. longum*, in protecting rats from OVX-induced bone loss. Additionally, this study evaluated the expression of a set of key genes related to bone metabolism.

## 2. Materials and Methods

### 2.1. Animal Model and Food Intake

Twenty-four 10-week-old female mature* Sprague*-*Dawley* rats, with a mean weight of 280–290 g, were obtained from the Faculty of Medicine and Health Sciences, Universiti Putra Malaysia (Serdang, Selangor, Malaysia). The rats were placed in plastic cages in a controlled environment animal facility at 22 ± 2°C with a 12-hour light/dark cycle. They were acclimatized for two weeks and given a standard rat chow pellet (Ridley Agri Products, Sydney, New South Wales, Australia) (Table 1) and* ad libitum* water access. The animal study was performed in an animal house at the Faculty of Medicine and Health Sciences, Universiti Putra Malaysia. Guidelines for the use of animals were strictly followed. The study was approved by the Animal Care and Use Committee (ACUC) of the Faculty of Medicine and Health Sciences, Universiti Putra Malaysia, with approval number UPM/FPSK/PADS/BR-UUH/00483.

### 2.2. Surgical Procedure and Grouping

The rats were weighed with a digital weighing scale (Marte-AS 2000C, Sao Paulo, Brazil) and, prior to the surgery, the body weight-matched rats were randomly divided into 3 groups with 8 rats in each group, namely, G1: sham-ovariectomized (sham), G2: ovariectomized (OVX), and G3: OVX +* Bifidobacterium longum* (OVX +* B. longum*). Either sham-OVX or OVX surgery was then performed on specific groups. Body weight was determined once a week. Before surgery, the rats were anesthetized using a combination of xylazine and ketamine 12 mg/80 mg/kg, injected intraperitoneally [22]. The same surgeon did all surgery procedures.

### 2.3. Probiotic Supplementation

After surgery, the rats were placed back in their cage for 2 weeks to recover. The rats in group 3 were then supplemented with 1 mL of* B. longum* (10^8^–10^9^) (CFU/mL), via oral gavage daily throughout the study (16 weeks), while sham and control groups received demineralized water daily. The amount of the bacteria used was based on a previous study [7]. The* B. longum* bacteria were purchased from the American Type Culture Collection (ATCC 15707; Rockville, MD, USA) and were activated at three continuous times in MRS broth (De Man Rogosa and Sharpe, Difco, Detroit, MI, USA) modified with 0.02% sodium carbonate, 0.01% calcium chloride dehydrate, and 1% solution of 0.05% L-cysteine as previously described [7].

### 2.4. Blood Biochemical Assay, Euthanasia, and Sampling

At the end of the intervention, the rats were anesthetized using a combination of xylazine and ketamine. The blood was collected via heart puncture and the animals were subsequently euthanized. The blood was placed into plain tubes and then centrifuged at 3,000 rpm for 10 min at 4°C, to obtain the serum for further analysis. The serum content of calcium and magnesium was measured using Roche Cobas C-311 Japan analyzer (Hitachi, Japan). OC and CTX were analyzed using standard commercial ELISA kit, Rat-MID OC EIA (IDS, Fareham, UK) for OC and RatLaps EIA (IDS) for CTX. The femurs were removed gently and preserved in 10% buffered formalin for further analyses. For RNA isolation purpose, the right femur was immediately transferred into RCL2 (Alpheys, Plaisir, France) and stored at −80°C until further assessment.

### 2.5. Evaluation of the Bone Mineral Content

Calcium, magnesium, and zinc content of the femur were determined by atomic absorption spectrophotometry (novAA 400; Analytik Jena, Jena, Germany) according to a previously described procedure [23] with slight modifications. Firstly, the femurs were dried at 105°C for 24 h and then placed in a muffle furnace at 550°C for another 24 h to obtain the ash samples. The ashes were then crushed and hydrolyzed with 6 M HCL. Calcium content was determined at a wavelength of 422.7 nm and lamp current 4.0 mA, zinc content was determined at a wavelength of 213.9 nm and lamp current 2.0 mA, and magnesium was determined at a wavelength of 285.2 nm and lamp current 2.0 mA.

### 2.6. Micro-CT (*μ*-CT) Analysis

Femur images of each rat were acquired after dissection using *μ*-CT (SkyScan, 1176, Brukler-Micro CT, Kontich, Belgium) at a resolution of 35 *μ*m, filter 0.5, exposure 100, voltage 40, and current 100. The correction of beam-hardening was performed to improve the quality of the image before scanning and during reconstruction. The femur images were then reconstructed with NRecon software version 1.6.3.3 and analyzed using SkyScan CT analyzer software version 1.9.1 to measure BMD (gr/cm^3^), the percentage of bone volume/total volume (BV/TV%), trabecular thickness (Tb.Th) (mm), trabecular number (Tb.N) (mm^−1^), trabecular separation (Tb.Sp) (mm), and the percentage of total porosity (to. pro%). Calibration of the *μ*-CT analyzer was performed with phantom material of calcium hydroxyapatite (CaHA) with a size of 4 mm in pairs, concentrations of 0.25 and 0.75 g/cm^3^, and their attenuation coefficient (AC).

### 2.7. Evaluation of the Femur Physical and Biomechanical Properties

The femur weight was measured using a digital electronic scale (Marte-AS 2000C, Sao Paulo, Brazil). The distance from the top edge of the head to the bottom edge as the femur length and the circumference of the midpoint length as the femur thickness were measured using a stainless-steel caliper. The femoral breaking force was assessed by using the three-point bending method according to Goda et al. [24] in the center of the femur using a universal testing machine (Instron Ltd., 8874, High Wycombe, UK), equipped with 5 KN load transducer.

### 2.8. Bone Histology Assessment

The formalin, in which the femurs were fixed, was periodically replaced until histology measurements were conducted. The first half of each left femur sample was allowed to decalcify in 5% nitric acid over 24 h. The samples were then transferred to an automated vacuum tissue processor (Leica ASP 300, Wetzlar, Germany) for dehydration. Samples were then embedded in paraffin histology wax and sectioned at 6 *μ*m thickness using a microtome (Leica, Wetzlar, Germany). At the final stage, the sectioned samples were stained with hematoxylin and eosin (H&E) and observed under a light microscope (Olympus BX51TRF-CCD Microscope, Tokyo, Japan). Static parameters, including osteoblast surface/bone surface (ObS/BS), osteoclast surface/bone surface (OcS/BS), eroded surface/bone surface (ES/BS), osteoid surface/bone surface (OS/BS), and osteoid volume/bone volume (OV/BV), were measured using a quantitative stereological method for histology, known as the Weibel technique [25]. All histomorphometric measurements were performed in the secondary spongy area, which is rich in trabecular. The selected region was located 1 mm from the lateral cortex and 3–7 mm from the lowest point of the growth plate [26]. Bone histomorphometric measurement was analyzed as recommended by the American Society of Bone Mineral Research (ASBMR) Histomorphometry Nomenclature Committee [26].

### 2.9. RNA Isolation and Real-Time PCR

RNA was extracted from the frozen right femur of the rats using the HiYield Total RNA Mini Kit (Real Biotech, Taipei, Taiwan) according to the manufacturer's instructions. RNA yield was then reported according to the absorbance at 260 nm using a NanoDrop ND-1000 UV-Vis (NanoDrop Technologies, Wilmington, DE, USA). The RNA quality was assessed by using the absorbance ratio at 260/280 nm along with the absorbance ratio of 260/230 nm. The extracted RNA was utilized only when the absorbance ratio at A260/230 and A260/280 showed readings between 1.8 and 2.0 with specific bands on gel electrophoresis [27]. Next, RNA was reverse transcribed to cDNA using i-script cDNA synthesis kit (Bio-Rad, Munich, Germany) according to the manufacturer's instruction. Real-time RT-PCR was performed in a reaction mixture containing 3 *μ*L of cDNA in a total volume of 25 *μ*L per reaction with the 2x SYBR Green I Hot-Start real-time PCR-Mix. Amplifications were then run in a Thermal Cycler using Eppendorf Realplex system (Eppendorf, Hamburg, Germany) with an initial denaturation at 95°C for 15 min, followed by 40 cycles of 95°C for 15 s, 58.6°C for 40 s, and 68°C for 20 s. Data were reported as fold changes relative to the housekeeping gene.

The forward and reverse primers were designed based on sequences obtained from the ENA and GenBank (http://www.ncbi.nlm.nih.gov/tools/primer-blast/) databases as follows:* Bmp-2,* accession number NM_017178, forward primer: CAGGTCTTTGCACCAAGATG, and reverse primer: GCTGGACTTAAGACGCTTCC;* Sparc*, accession number NM_012656, forward primer: CAGGTGGAAATGGGAGAGTT, and reverse primer: GTTTGCAATGATGGTTCTGG; and glyceraldehyde-3-phosphate dehydrogenase (GADPH) as a housekeeping gene, accession number NM_017008, forward primer: TCAAGAAGGTGGTGAAGCAG, and reverse primer: AGGTGGAAGAATGGGAGTTG. The primers were supplied by Helix Biotech (Helix Biotech Ltd., Richmond, British Columbia, Canada). The primers were then diluted to a final concentration of 100 *μ*L with nuclease-free water.

### 2.10. Enumeration of Bifidobacteria in the Feces

After probiotic supplementation, the fresh feces of the rats were cultivated in Bifidus Selective Medium agar (BSM agar, Fluka, Buchs, Switzerland) and bifidobacteria were enumerated after incubation. The plates of cultivated bacteria were placed for 72 h at 37°C in an anaerobic jar to provide anaerobic conditions by gas pack (Oxoid Ltd., Basingstoke, UK). After the incubation period, the colonies were counted using the colony-counting device (Stuart Scientific, Staffordshire, UK) and the number of colonies was expressed as log_10_⁡ CFU/g.

### 2.11. Statistical Analysis

The data were checked for normality using the UNIVARIATE procedure of SAS software (version 8.2, SAS Institute Inc., Cary, NC, USA). Microbial data were converted to log_10_ and reported as a wet weight basis. Data were analyzed by one-way analysis of variance (ANOVA) with PROC MIXED in SAS. The results were expressed as mean ± SEM and the differences among the groups were analyzed with a post hoc Tukey's test. Significance was considered at *p* < 0.05.

## 3. Results

### 3.1. The Effect of* B. longum* on Body Weight

At the end of the study, the body weight of the rats from the sham, OVX, and* B. longum*-fed groups was 349.5 ± 29.1 g, 385.1 ± 28.5 g, and 381.1 ± 29.3 g, respectively. No significant difference was observed between the groups (*p* > 0.05).

### 3.2. The Effect of* B. longum* on Blood Biochemical Assay and Bone Mineral Content

The serum parameters are summarized in Table 2. The OVX rats without* B. longum* supplementation showed a significant reduction in serum osteocalcin (OC; 76.81 ± 6.44; *p* < 0.05) compared to the sham (184.61 ± 6.93; *p* < 0.05) and* B. longum*-supplemented groups (101.31 ± 9.21; *p* < 0.05). Ovariectomy increased serum level of CTX compared to the sham group (249.28 ± 7.57 over 113.77 ± 20.02; *p* < 0.05), which indicated an increased bone resorption activity. However, the* B. longum*-supplemented group showed a decrease in CTX serum level (147.05 ± 6.25; *p* < 0.05) compared to the OVX group. Ca and Mg serum levels (Table 2) and the femur mineral content, including Ca, Mg, and Zn (mg/g) (Table 3), were not significantly affected by* B. longum* supplementation (*p* > 0.05).

### 3.3. *B. longum* Increased BMD and Changed the Trabecular Structure of the Femur

**Figure 1:**
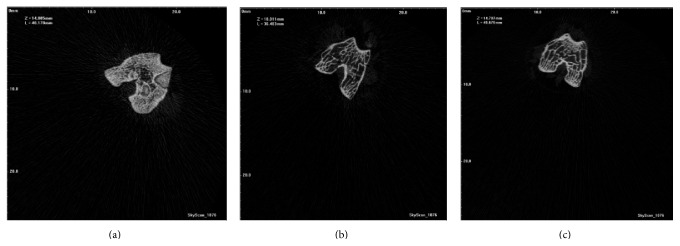
Reconstruction of *μ*-CT scan image of the femur in the different groups. (a) Sham: sham-ovariectomized; (b) OVX: ovariectomized; (c) OVX + 1 mL of 10^8^–10^9^ CFU of* B*.* longum*.

The reconstruction of *μ*-CT scan images of the femur is illustrated in Figure 1. BMD (g/cm^3^), BV/TV (%), total porosity (%), Tb.N (mm^−1^), Tb.Th (mm), and Tb.Sp (mm) were measured using the *μ*-CT analyzer and presented in Table 4. OVX decreased the percentage of BV/TV (49.60 ± 5.69) and Tb.N (0.10 ± 0.01) (*p* < 0.05) and increased the total porosity percentage (44.25 ± 3.98; *p* < 0.05). The* B. longum* supplementation increased the BV/TV% and Tb.N and decreased total porosity %, abolishing the effect of OVX. In addition,* B. longum* supplementation increased the Tb.Th close to that of the sham group. In this regard, OVX group showed the least Tb.Th as compared to the sham and* B. longum*-fed group (4.83 ± 0.27, 7.33 ± 0.657, and 7.21 ± 0.48, resp.; *p* < 0.05). The present supplementation also increased the BMD of the femur compared to the OVX group (0.89 ± 0.06 versus 0.59 ± 0.07; *p* < 0.05). There was no significant change in Tb.Sp of the femur between groups.

### 3.4. Effect of* B. longum* on the Femur Physical and Biomechanical Properties

**Figure 2:**
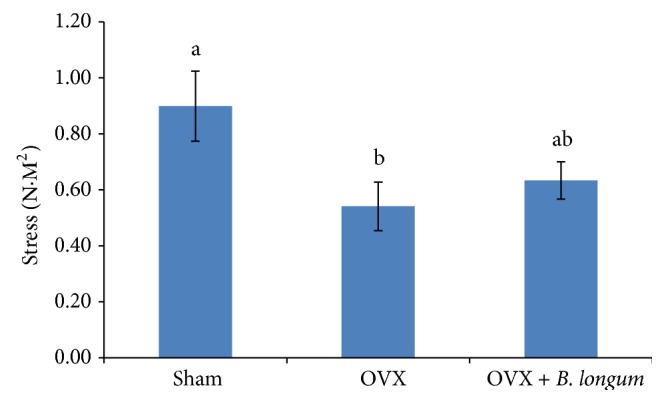
Effect of* B. longum* on the rat femur stress. Ten-week-old female* Sprague*-*Dawley* rats were supplemented with 1 mL* B. longum* (10^8^–10^9^ CFU) once a day for 16 weeks, starting 2 weeks after surgery, while sham and OVX groups received 1 mL of demineralized water. At the end of the study, the femoral breaking force of dissected femurs was tested by using the three-point bending method in the center of the femur using a universal testing machine. Values represent the mean ± SEM (*n* = 8 in each group). ^ab^Values with different letters in the same row are significantly different at *p* < 0.05 based on one-way ANOVA, followed by Tukey's post hoc test.

The femur weight, height, and thickness did not differ significantly between the groups (Table 5). However, OVX reduced the strength of the femur (0.54 ± 0.09) as compared to that of the sham group (0.90 ± 0.12; *p* < 0.05). The* B. longum* supplementation increased the strength of the femur (0.63 ± 0.07; *p* < 0.05) (Figure 2).

### 3.5. Effect of* B. longum* on Osteoblasts, Osteoclasts, Osteoid, and Eroded Surface over Bone Surface of the Femur

**Figure 3:**
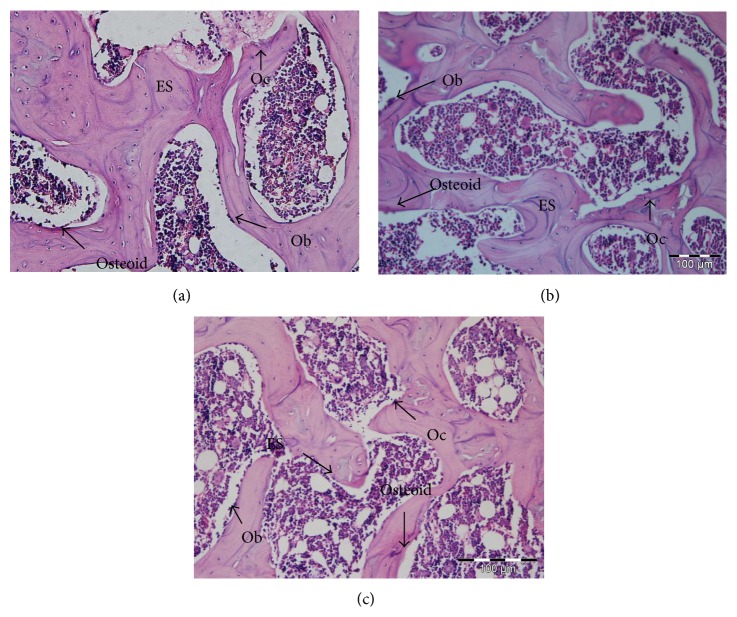
H&E stained sections of the femur. (a) Sham: sham-ovariectomized; (b) OVX: ovariectomized; (c) OVX + 1 mL of 10^8^–10^9^ CFU of* B. longum*.

Osteoclasts, osteoblasts, and osteoid cells as well as eroded surface are shown in Figure 3. In addition, histomorphometric results, including ObS/BS, OcS/BS, ES/BS, OS/BS, and OV/BV, are summarized in Table 6. Compared to the sham group, OVX decreased ObS/BS (37.05 ± 0.44), OS/BS (18.08 ± 0.17), and OV/BV (7.06 ± 0.30) and increased OcS/BS (27.96 ± 0.33) and ES/BS (37.78 ± 0.30) (*p* < 0.05).* B. longum* supplementation significantly increased the ObS/BS (46.85 ± 1.42), OS/BS (23.06 ± 1.19), and OV/BV (10.15 ± 1.19) and decreased OcS/BS (25.54 ± 1.23) and ES/BS (34.24 ± 1.25) when compared to the OVX group (*p* < 0.05).

### 3.6. Effect of* B. longum* on the Expression of* Bone Morphometric Protein-2* (*Bmp-2*) and* Secreted Protein Acidic and Rich in Cysteine* (*Sparc*) Genes

**Figure 4:**
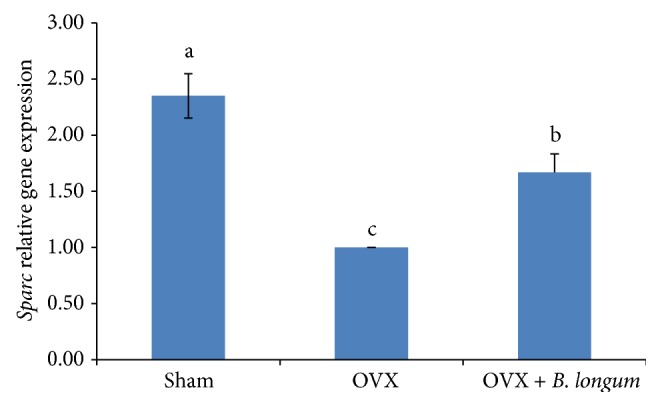
Effect of* B. longum* on* Sparc* gene expression. Ten-week-old female* Sprague*-*Dawley* rats were supplemented with 1 mL* B. longum* (10^8^–10^9^ CFU) once a day for 16 weeks, starting 2 weeks after surgery, while sham and OVX groups received 1 mL of demineralized water. At the end of the study,* Sparc* expression was quantified by reverse transcription-polymerase chain reaction (RT-PCR) analysis. Values represent the mean ± SEM (*n* = 8 in each group). ^abc^Values with different letters are significantly different at *p* < 0.05 based on one-way ANOVA, followed by Tukey's post hoc test.

**Figure 5:**
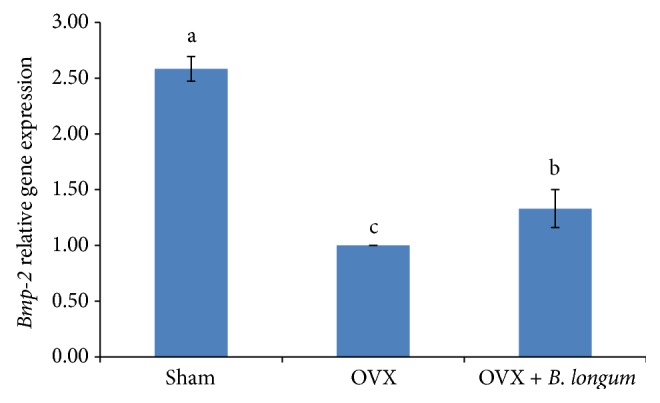
Effect of* B. longum* on* Bmp-2* gene expression. Ten-week-old female* Sprague*-*Dawley* rats were supplemented with 1 mL* B. longum* (10^8^–10^9^ CFU) once a day for 16 weeks, starting 2 weeks after surgery, while the sham and OVX groups received 1 mL of demineralized water. At the end of the study,* Bmp-2* expression was quantified by RT-PCR. Values represent the mean ± SEM (*n* = 8 in each group). ^abc^Values with different letters are significantly different at *p* < 0.05 based on one-way ANOVA, followed by Tukey's post hoc test.

*Sparc* and* Bmp-2* gene expression is shown in Figures 4 and 5.* Sparc* and* Bmp-2* were significantly downregulated in the OVX group (*p* < 0.05) and* B. longum* supplementation significantly upregulated the expression of* Sparc* (1.67 ± 0.16 fold changes; *p* < 0.05) and* Bmp-2* (1.33 ± 0.17 fold changes; *p* < 0.05) compared to that of the OVX group.

### 3.7. Enumeration of Bifidobacteria in Fecal Samples

**Figure 6:**
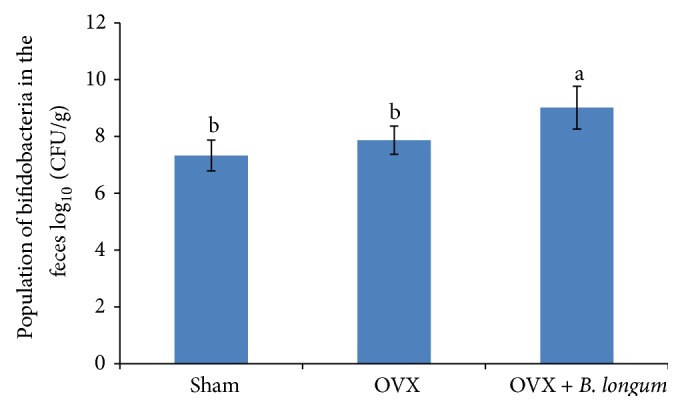
Effect of* B. longum* on the total population of bifidobacteria in the rat feces. Ten-week-old female* Sprague*-*Dawley* rats were supplemented with 1 mL* B. longum* (10^8^–10^9^ CFU) once a day for 16 weeks, starting 2 weeks after surgery, while the sham and OVX groups received 1 mL of demineralized water. At the end of the study, the amount of bifidobacteria was quantified in the feces. Values represent the mean ± SEM (*n* = 8 in each group). ^ab^Values with different letters are significantly different at *p* < 0.05 based on one-way ANOVA, followed by Tukey's post hoc test.

The total bifidobacteria count in the feces is shown in Figure 6.* B. longum* supplementation significantly increased the number of the total bifidobacteria (CFU/g) in the feces of the supplemented group (*p* < 0.05).

## 4. Discussion

At the end of the study, all groups showed a similar body weight, as expected since the three diets used in this study were isocaloric and food consumption was similar in all groups. This result is in agreement with a previous study from Bryk et al. [28]. In this study,* B. longum* could increase the number of bifidobacteria in the feces of the rats and increased bone mass density, suggesting that the preventative effects of* B. longum* on osteoporosis might be due to its probiotic effects.

Osteoporosis is accompanied with low BMD and changes in microstructure of bone tissue such as reduction in Tb.Th, Tb.N, and BV/TV% and increase in Tb.Sp, total porosity, and risk of bone fracture. These characteristics of osteoporosis were observed in the OVX non-supplemented group in the present study. Similar results were reported in previous studies [29, 30].* B. longum* supplementation decreased Tb.Sp and total porosity of the femur and, in parallel, increased Tb.N, Tb.Th, and BV/TV and led to the improvement of the femur BMD. Indeed, trabecular microstructure and BV/TV are the main determinants of bone fracture and bone strength and have a positive relation with bone loss [31]. The reduction of bone strength decreased bone elasticity and loss of bone elasticity is associated with bone fracture [32]. Thus, Tb.Th, Tb.N, and Tb.Sp as well as that of BV/TV are some of the predictors for bone fracture risk and osteoporosis, which were modulated by* B. longum* supplementation in this study. In fact, similar results regarding the changes of the trabecular microstructure by probiotics consumption were also reported by Chiang and Pan [7] and Shim et al. [33].

In the present study,* B. longum* reduced the bone resorption and increased bone formation as indicated by increasing OC and decreasing CTX levels in the serum and changes in the ratio of ObS/BS, OS/BS, OcS/BS, and ES/BS. A continuous bone remodeling process requires both osteoblasts and osteoclasts. Osteoclasts dissolve the bone matrix, which results in the occurrence of eroded surface [1], but the bone formation proceeds concurrently through the help of osteoblasts. Serum CTX is produced by osteoclasts during the bone resorption process and is a bone resorption marker, while serum OC is secreted only by osteoblast and osteocytes cells during the bone formation and is a bone formation marker [34]. Thus, the rise in the bone resorption process is accompanied with the rise of osteoclast activity and increased levels of CTX in the serum, while a rise in the bone formation process is accompanied with the increase of osteoblast activity and OC levels in the serum [35, 36]. Therefore, the activity of osteoclasts and osteoblasts cells can be evaluated by measuring OC and CTX in the serum [37] and assessing the ratio of osteoblasts and osteoclast surface over bone surface. A rise in serum CTX and the ratio of OSc/BS and ES/BS in the trabecula, which occurred in the OVX rats, indicated that the bone resorption activity precedes the bone formation process. In the present study,* B. longum* supplementation decreased the ratio of OSc/BS and ES/BS and serum levels of CTX, while it increased the ratio of ObS/BS, OS/BS, and OcS/BS and serum levels of OC, indicating its beneficial health effect as a probiotic in preventing osteoporosis.

In addition,* B. longum* supplementation increased* Bmp-2* and* Sparc* expression.* Bmp-2* is a growth factor and plays an essential role in skeletal development, especially in early embryogenesis [38], and is well known for bone formation signal [39] and appears as a key agent in the osteoblastic differentiation [40]. Besides,* Bmp-2* is a gene that controls both the proliferation and differentiation of osteoblasts [41]. The rise in* Bmp-2* expression resulted in an increase in* Bmp-2* secretion in cells. Therefore, osteoblasts can regulate their own proliferation and differentiation, which is a key role during bone remodeling. On the other hand,* Bmp-2* can also help bone regeneration and repair and prevent apoptosis [42]. Bone regeneration is related to the elasticity of the bone and is associated with bone strength [43]. Therefore, the increasing femur strength by probiotic supplementation in the current study may also relate to the higher expression of* Bmp-2*. In this regard Kanakaris et al. [40] showed treatment of osteoporotic fracture with* Bmps* induced rapid increase of bone strength in entire skeleton, especially at area of bone fracture. Another gene investigated in this study is* Sparc*, which encodes a noncollagenous protein in extracellular bone matrix necessary for collagen calcification in the bone [44]. Recent study investigated therapeutic effect of* Sparc* for treating or preventing bone related diseases. It is related to tissue remodeling, repair development, and bone mineralization [45]. Since* Sparc* participates in bone remodeling and is a Ca-binding receptor, and helps bone calcification [46], increasing its expression leads to increased bone calcification, resulting in stronger bones as observed in OVX rats supplemented with* B. longum*. Thus,* Sparc* upregulation can explain the increase in bone strength induced by* B. longum* supplementation.

An increased number of bifidobacteria in the feces of the rats demonstrated the increased colonization of the intestine and cecum by these bacteria and suggested the bacterial cell wall was intact after passage away through gastrointestinal tract (GI) and the bifidobacteria used in this study are resistant to acids and bile of the GI [47]; this helps the bacteria to survive through the passage of the GI in order to exert a better function [48]. Generally, probiotics have significant effects on the digestive tract by improving proliferation of intestinal epithelial cells due to the enhancement of short chain fatty acids (SCFA) production [49]. The production of SCFA decreases luminal pH, subsequently increases mineral absorption [50] via their solubilization, and reduces secondary acid formation in the colon [17]. Thus, Ca absorption from the intestine increases due to the survival and functionality of probiotics in the GI. In this study,* B. longum* supplementation induced an increase in serum Ca levels, upregulated* Sparc*, and increased the Ca content of the femur as compared to the OVX non-supplemented group. Thereby,* B. longum* supplementation as a probiotic ameliorated bone loss induced by OVX. In addition, some studies showed chemotherapy in rats showed bone loss effects [51, 52], since our results showed that the* Bifidobacterium longum* could prevent the bone loss in the rat; thus it can be applicable for the rats under chemotherapy.

This research was not without its weaknesses. In this regard, there were some limitations that can be discussed. Firstly, this research only assessed BMD and BMC of the rats at the end of the study and compared between different groups. The study did not measure BMD and BMC of the rats before starting the treatments to compare changes of BMD and BMC of the femur before and after intervention. This limitation occurred due to the lack of available instrument before starting the intervention. Second, this research did not design the sham group supplemented with probiotic as a control for comparison. We just confine two groups as a control and one intervention group.

## 5. Conclusion

*B. longum* supplementation prevents bone loss induced by OVX in rats. Our findings illustrated that* B. longum,* as a probiotic supplement, increased bone formation, decreased bone resorption, and changed the microstructure of the femur. The femur BMD was increased due to the upregulation of* Sparc* and* Bmp-2* genes. These data suggest that* B. longum* should be considered as a potential therapeutic agent to prevent postmenopausal osteoporosis. However, additional studies are required to explain the mechanism by which* B. longum* supplementation affects the outcomes described in this study.

## Figures and Tables

**Table 1:** Nutrients composition of rat chow diet.

Standard rat chow diet ingredient	
Crude protein	21.0%
Crude fiber	7.3%
Crude fat	3.0%
Moisture	13.0%
Ash	8.0%
Calcium	1.0%
Phosphorus	0.5%
Nitrogen-free extract	49.0%

**Table 2:** Effect of *B. longum* on blood serum parameters.

Serum parameters	Sham	OVX	OVX + *B. longum*	*p* value
OC (ng/mL)	184.61 ± 6.93^a^	76.81 ± 6.44^c^	101.31 ± 9.21^b^	0.001
CTX (ng/mL)	113.77 ± 20.02^b^	249.28 ± 7.57^a^	147.05 ± 6.25^b^	0.009
Ca (mmol/L)	2.27 ± 0.12	2.23 ± 0.12	2.27 ± 0.06	0.939
Mg (mmol/L)	0.97 ± 0.07	0.89 ± 0.09	0.91 ± 0.06	0.751

Ten-week-old female *Sprague-Dawley* rats were supplemented with 1 mL of *B. longum* (10^8^–10^9^ CFU) once a day for 16 weeks, starting 2 weeks after surgery, while sham and OVX groups received 1 mL of demineralized water. At the end of the study, the blood serum of the rats was analyzed for osteocalcin (OC), C-terminal telopeptide (CTX), calcium (Ca), and magnesium (Mg). Values represent the mean ± standard error of the mean (SEM) (*n* = 8 in each group).

^abc^Values with different letters in the same row are significantly different at *p* < 0.05 based on one-way ANOVA, followed by Tukey's post hoc test.

**Table 3:** Effect of *B. longum* on the mineral content of the femur.

Femur mineral content (mg/g)	Sham	OVX	OVX + *B. longum*	*p* value
Ca	246.23 ± 10.14	242.09 ± 5.16	242.45 ± 15.26	0.280
Mg	33.23 ± 0.15	32.44 ± 0.12	32.76 ± 0.31	0.220
Zn	17.23 ± 2.04	13.89 ± 0.67	13.96 ± 1.25	0.241

Ten-week-old female *Sprague-Dawley* rats were supplemented with 1 mL of *B. longum* (10^8^–10^9^ CFU) once a day for 16 weeks, starting 2 weeks after surgery, while sham and OVX groups received 1 mL of demineralized water. At the end of the study, the calcium (Ca), magnesium (Mg), and zinc (Zn) content of the femur was analyzed. Values represent the mean ± SEM (*n* = 8 in each group).

No significant difference was observed between the groups based on one-way ANOVA, followed by Tukey's post hoc test.

**Table 4:** Effect of *B. longum* on the microstructure and BMD of the femur.

Parameters	Sham	OVX	OVX + *B. longum*	*p* value
BV/TV (%)	73.66 ± 6.45^a^	49.60 ± 5.69^b^	60.55 ± 3.57^ab^	0.025
Porosity (%)	24.84 ± 5.45^b^	44.25 ± 3.98^a^	34.85 ± 2.25^ab^	0.026
Tb.Th (mm)	7.33 ± 0.65^a^	4.83 ± 0.27^b^	7.21 ± 0.48^a^	0.004
Tb.Sp (mm)	5.48 ± 0.42	5.30 ± 0.37	6.03 ± 0.16	0.234
Tb.N (mm^−1^)	0.12 ± 0.01^a^	0.10 ± 0.01^b^	0.09 ± 0.01^ab^	0.040
BMD (g/cm^3^)	1.06 ± 0.02^a^	0.59 ± 0.07^b^	0.89 ± 0.06^a^	0.013

Ten-week-old female *Sprague-Dawley* rats were supplemented with 1 mL of *B. longum* (10^8^–10^9^ CFU) once a day for 16 weeks, starting 2 weeks after surgery, while sham and OVX groups received 1 mL of demineralized water. At the end of the study, dissected femurs were analyzed with a micro-CT scan analyzer. Values represent the mean ± SEM (*n* = 8 in each group).

BV/TV: bone volume/total volume; Tb.Th: trabecular thickness; Tb.Sp: trabecular separation; Tb.N: trabecular number; BMD: bone mass density.

^ab^Values with different letters in the same row are significantly different at *p* < 0.05 based on one-way ANOVA, followed by Tukey's post hoc test.

**Table 5:** Effect of *B. longum* on the physical properties of the femur.

Femur physical characteristics	Sham	OVX	OVX + *B. longum*	*p* value
Weight (g)	1.06 ± 0.03	1.09 ± 0.05	1.10 ± 0.04	0.842
Height (mm)	37.28 ± 0.75	37.36 ± 0.41	37.37 ± 0.22	0.989
Thickness (mm)	4.05 ± 0.16	4.17 ± 0.09	4.17 ± 0.07	0.721

Ten-week-old female *Sprague-Dawley* rats were supplemented with 1 mL of *B. longum* (10^8^–10^9^ CFU) once a day for 16 weeks, starting 2 weeks after surgery, while sham and OVX groups received 1 mL of demineralized water. At the end of the study, the femur weight was measured by using a digital electronic scale and the height and thickness were measured using a stainless-steel caliper. Values represent the mean ± SEM (*n* = 8 in each group).

No significant difference was observed between the groups based on one-way ANOVA, followed by Tukey's post hoc test.

**Table 6:** Effect of *B. longum* on histomorphometric measurements of the femur.

Parameters (%)	Sham	OVX	OVX + *B. longum*	*p* value
ObS/BS	53.30 ± 2.29^a^	37.05 ± 0.44^c^	46.85 ± 1.42^b^	0.046
OcS/BS	23.01 ± 1.34^c^	27.96 ± 0.33^a^	25.54 ± 1.23^b^	0.001
OS/BS	24.02 ± 1.27^a^	18.08 ± 0.17^c^	23.06 ± 1.19^b^	0.034
OV/BV	13.33 ± 1.28^a^	7.06 ± 0.30^c^	10.15 ± 1.19^b^	0.004
ES/BS	28.97 ± 2.15^c^	37.78 ± 0.30^a^	34.24 ± 1.25^b^	0.001

Ten-week-old female *Sprague-Dawley* rats were supplemented with 1 mL *B. longum* (10^8^–10^9^ CFU) once a day for 16 weeks, starting 2 weeks after surgery, while sham and OVX groups received 1 mL of demineralized water. At the end of the study, the percentage of osteoblast surface/bone surface (ObS/BS), osteoclast surface/bone surface (OcS/BS), eroded surface/bone surface (ES/BS), osteoid surface/bone surface (OS/BS), and osteoid volume/bone volume (OV/BV) was measured from H&E stained sections of the dissected femur. Values represent the mean ± SEM (*n* = 8 in each group).

^abc^Values with different letters in the same row are significantly different at *p* < 0.05 based on one-way ANOVA, followed by Tukey's post hoc test.
